# Worth living or worth dying? The views of the general public about allowing disabled children to die

**DOI:** 10.1136/medethics-2019-105639

**Published:** 2019-10-15

**Authors:** Claudia Brick, Guy Kahane, Dominic Wilkinson, Lucius Caviola, Julian Savulescu

**Affiliations:** 1 Monash University Faculty of Medicine Nursing and Health Sciences, Clayton, Victoria, Australia; 2 Oxford Uehiro Centre for Practical Ethics, Faculty of Philosophy, University of Oxford, Oxford, UK; 3 John Radcliffe Hospital, Oxford, UK; 4 Murdoch Childrens Research Institute, Melbourne, Victoria, Australia; 5 Department of Experimental Psychology, University of Oxford, Oxford, UK

**Keywords:** Allocation of Health Care Resources, Clinical Ethics, Ethics, Quality/Value of Life/Personhood, End-of-life

## Abstract

**Background:**

Decisions about withdrawal of life support for infants have given rise to legal battles between physicians and parents creating intense media attention. It is unclear how we should evaluate when life is no longer worth living for an infant. Public attitudes towards treatment withdrawal and the role of parents in situations of disagreement have not previously been assessed.

**Methods:**

An online survey was conducted with a sample of the UK public to assess public views about the benefit of life in hypothetical cases similar to real cases heard by the UK courts (eg, Charlie Gard, Alfie Evans). We then evaluated these public views in comparison with existing ethical frameworks for decision-making.

**Results:**

One hundred and thirty participants completed the survey. The majority (94%) agreed that an infant’s life may have no benefit when well-being falls below a critical level. Decisions to withdraw treatment were positively associated with the importance of use of medical resources, the infant’s ability to have emotional relationships, and mental abilities. Up to 50% of participants in each case believed it was permissible to either continue *or* withdraw treatment.

**Conclusion:**

Despite the controversy, our findings indicate that in the most severe cases, most people agree that life is not worth living for a profoundly disabled infant. Our survey found wide acceptance of at least the permissibility of withdrawal of treatment across a range of cases, though also a reluctance to overrule parents’ decisions. These findings may be useful when constructing guidelines for clinical practice.

## Introduction

Decisions to limit life-sustaining treatment and allow a child to die are common in paediatric intensive care units and widely ethically accepted.^1–3^ The vast majority of paediatric treatment limitation decisions in intensive care units worldwide are made through consensus between physicians, patients and their families. However, as with any life-and-death issue, ethical issues are prominent and disagreement is not uncommon—and perhaps on the rise.^4 5^ The increasing capacity of medicine to keep children alive and the increasingly availability of information via the internet and social media means doctors and families may reach different conclusions about whether or when to discontinue life-sustaining treatment.^4^ This has led to multiple high-profile court cases, such as that of Charlie Gard in 2017 (box 1). This case did not occur in isolation: other high profile verdicts include those of Child MB in 2006, Charlotte Wyatt in 2004 and Alfie Evans in 2018.^6–8^

Box 1 **The Charlie Gard case**

Charlie Gard was a few months old when he developed progressive muscle weakness and was diagnosed with a rare genetic disorder: Mitochondrial DNA Depletion Syndrome. Soon after, Charlie was completely paralysed, deaf, had organ dysfunction and was dependent on mechanical ventilation at Great Ormond Street Hospital. Physicians believed that further treatment was futile and life support should be withdrawn, while Charlie’s parents had located a US-based specialist who was prepared to trial an experimental treatment; the parents thus wanted ventilation to continue. Six months of legal proceedings ensued, with hearings at the High Court, the Court of Appeal, Supreme Court and European Court of Human Rights all judging in favour of the physicians, ruling that continued treatment would not be in Charlie’s best interests, and that his ‘current quality of life is not one that should be sustained’.^4 38–40^ The cases attracted high levels of media and public attention, and garnered opinions from as far afield as the Vatican and the White House.

### A life not worth living

Central to these cases of disagreement is the concept that in some situations, a child’s degree of disability, severity of illness and/or burden of medical treatment are so great that it would be best to allow them to die. We define the concept as below:

A life not worth living (LNWL): A life in which future burdens for the individual outweigh benefits. There is negative net future well-being.^9^

It is important to clarify that this concept encompasses the future prudential value of the life to the individual concerned (ie, it is not about the value of an individual to others).^1^

### UK legal perspective

Though medical guidelines have been published to aid physicians in these decisions, the most important principles in the UK have evolved through common (case) law. Because infants have never had decision making capacity or known treatment preferences, these decisions must bypass the informed consent and substituted judgement standards that are first turned to in equivalent adult cases.^10^ Instead, treatment withdrawal is based on a best interests standard,^11^ which has been described as encompassing ‘medical, emotional and all other welfare issues’ from ‘the assumed point of view of the child’.^7^ A judgement may then be made that, due to a prognosis of severe future disability, life would not be worth living.^12^ This was further elaborated in the 2006 case of Child MB, when Justice Holman stated that the value of life ‘may be outweighed if the pleasures and the quality of life are sufficiently small and the pain and suffering or other burdens of living are sufficiently great’.^6^ Although the financial cost of treatment may be significant in clinical practice, resources have been considered irrelevant to these legal decisions.^2^

### Ethical perspective

In answering whether a life is worth living, ethicists have frequently turned to the theories of well-being, which ask what makes someone’s life go best: these range from subjective views such as hedonism, where pleasure and pain alone are valued,^13^ to objective views, where other goods (such as knowledge, autonomy and relationships) are also considered dimensions of well-being.^14^ There is a further question of how these well-being judgements should map to treatment decisions. The traditional way of viewing this, as described in the legal judgements, has been labelled a ‘Zero Line View’,^9 15^ where the point at which treatment must be withdrawn is the same point at which life has no benefit. However, given significant medical prognostic uncertainty about the quality of future life and moral uncertainty about what level of life is worth living, it is extremely difficult to precisely define where the zero line is.

**Figure 1:**
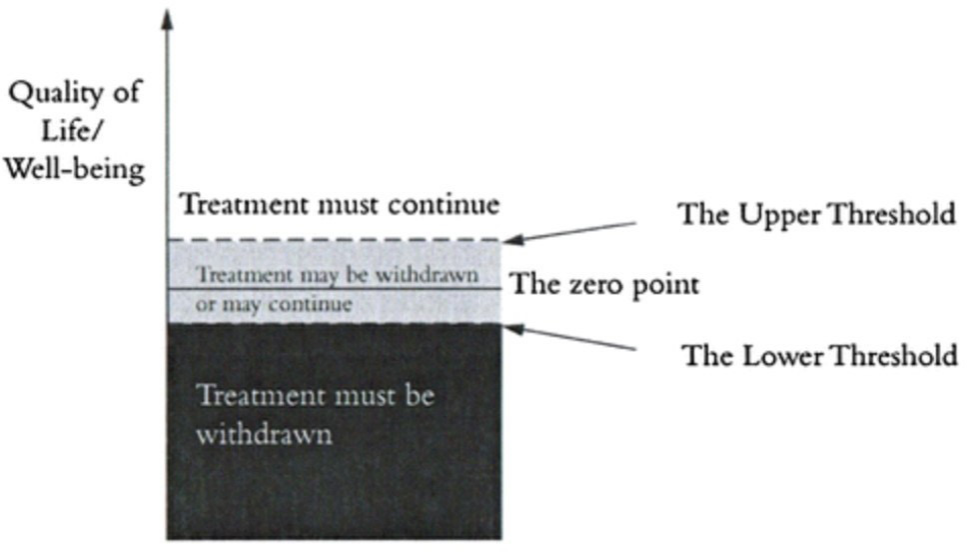
The Threshold Framework for treatment withdrawal.^16^ Reproduced with permission from *Death or Disability? The ‘Carmentis Machine’ and decision-making for critically ill children.*

An alternative to the Zero Line View is the Threshold View.^9 16^ The threshold view defines an upper and lower threshold, where above the top threshold it is ethically obligatory to continue or provide treatment, and below the lower threshold, it is obligatory to withdraw/withhold treatment (figure 1). When future well-being lies between the two thresholds, it would be permissible to *either* continue or withdraw treatment. Although the threshold view permits treatment withdrawal for some infants who might have had lives worth living, Wilkinson has previously argued that this framework is preferable because it recognises the uncertainty present in these decisions, as well as the significant interests of family members due to the future burden of care of an infant or child with severe impairment.^9 16^

### Empirical perspective

The media attention to the Gard case and substantial social media support for the family may suggest that the public are opposed to legal decisions like those made for Charlie Gard.^17^ However, there is no published data on public attitudes to such cases, on general understanding of the concept of a LNWL in infants or on what grounds people would support treatment withdrawal, if at all. It is unknown whether decisions on these questions are influenced by the presence of pain and suffering (in line with hedonism) or the lack of capacity for human relationships (in line with objective list views) or simply by considerations relating to scarce resources or parental autonomy.

Existing studies have assessed the views of the public and health professionals towards treatment withdrawal in the setting of adult patients in persistent vegetative state, finding that 66%–92% would support treatment withdrawal.^18–21^ Studies of public attitudes to cases involving children found strong support for parental autonomy in treatment choices, with one study reporting that a large proportion of participants would support parental requests for substandard treatment even at an increased risk of death for the child.^22^

However, these studies do not directly shed any light on what kind of life the public regards as not worth living, and they leave open many questions about public views towards withdrawal of treatment in severely ill infants. The aim of the present study was thus to assess public intuitions towards cases similar to those that have been adjudicated by the UK courts and to gauge their responses to specific ethical questions. Although empirical findings about public opinions cannot provide normative answers to what constitutes a LNWL, such data may be valuable for debate about how these decisions should be made, both in clinical practice and the legal sphere.

## Methods

Members of the UK public were recruited via an online survey platform developed for social science research, Prolific Academic and paid at a minimum rate set by Prolific of 5GBP an hour. Prolific has a diverse pool of participants and has been showing to produce high quality data equivalent to that of Amazon’s Mechanical Turk (MTurk).^23 24^ Unlike MTurk, however, Prolific Academic is not restricted to US citizens, thereby allowing for recruitment of UK-based participants, who make up 49% of the Prolific population and are more likely to be culturally familiar with the health and judicial systems in the UK.^25^ Inclusion criteria included being a UK resident and at least 18 years of age. Only participants with an approval rate of >95% on>10 previously completed surveys could participate. Participants were excluded if they did not complete the survey or failed any of three Instructional Manipulation Checks assessing attention and comprehension.^26^ Ethics approval was obtained from the Social Sciences & Humanities Inter-Divisional Research Ethics Committee of the University of Oxford.

### Section 1

The survey consisted of three sections. Section 1 consisted of six scenarios of severely ill hospitalised infants where withdrawal of life-sustaining treatment might be considered, and participants were asked about their view on the benefit of life for the infant and if they believed withdrawal of treatment would be justified. A brief explanation was given of these decisions and of the provision of mechanical ventilation and artificial nutrition and hydration. Four of the scenarios were simplified versions of the real-life cases of Charlie Gard, Alfie Evans, Charlotte Wyatt and Baby MB, derived from the conclusions of the judges and medical experts in transcripts of the trials. The remaining two cases were hypothetical scenarios constructed to reflect key standards assumed in legal cases and medical guidelines.^2 27^ The cases were standardised to ensure participants would not know which were hypothetical, and were presented on separate pages in random order to control for order effects.^28^ Participants were asked to ignore considerations of resources and the effect on family members in order to focus exclusively on the well-being of the infant.

**Figure 2:**
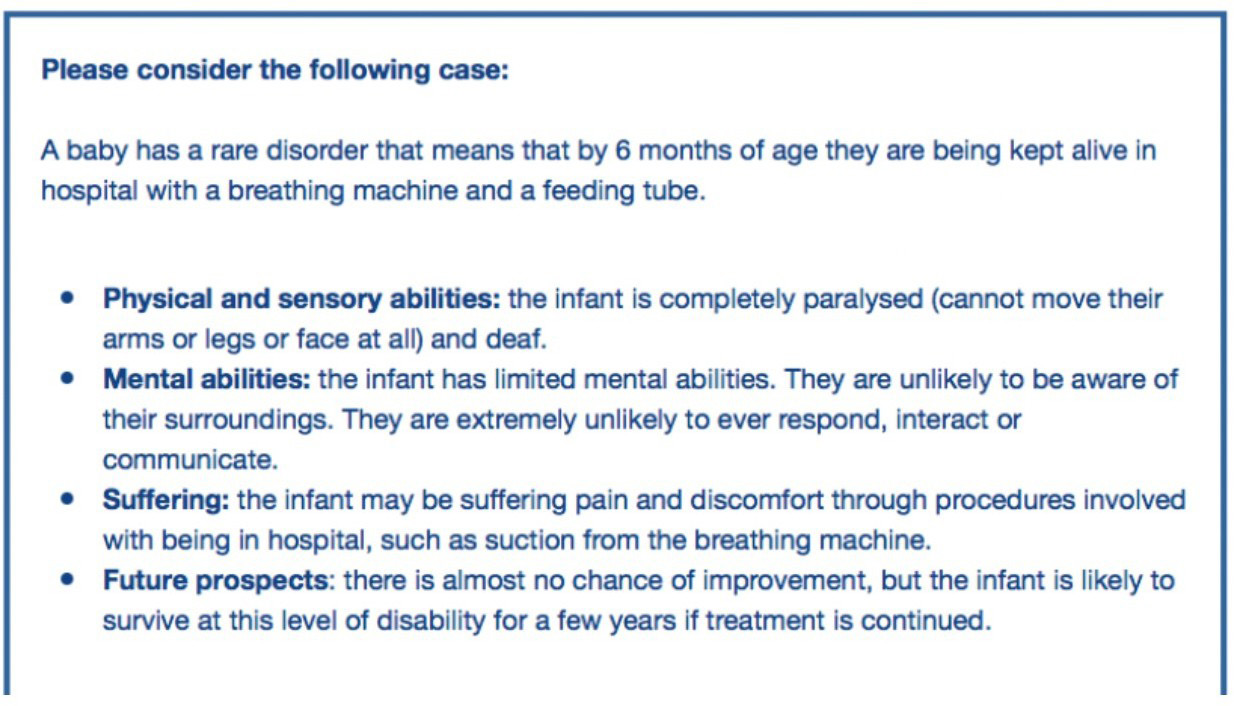
Example case from the survey: possible awareness.

The six cases used structured descriptions delineating in lay language each infant’s physical and sensory abilities, cognitive capacity, level of suffering and future prospects (example in figure 2).

Cognition ranged from unconsciousness or basic awareness alone to the potential for simple thoughts, emotions, interactions and relationships with parents, through to presumed normal cognition (table 1). For the purposes of this paper, the cases will be referred to by the labels described in table 1.

**Table 1:** Summary of key prognostic variables for each case scenario

Applicable to all cases: Approximately 6 months to 18 months of age—‘an infant’. Hospitalised and reliant on mechanical ventilation and artificial nutrition and hydration (except for case 6). No hope of improvement from this level of disability. Likely survival at this level of disability for a few years if treatment continued. All described in the survey in lay language with more complete descriptions of the illness and its consequences for the infant.			
Cases	Cognition	Suffering	Pleasure
Unaware: based on Alfie Evans	No cognition	No suffering	No pleasure
Possible Awareness: based on Charlie Gard	Possible awareness	Possible suffering	No pleasure
Minimal Cognition	Aware No interaction	Low suffering	Mild pleasures
Locked In: based on baby MB	Aware Cognitively intact but unable to communicate or interact due to paralysis	Medium suffering	Medium pleasures
Possible Relational Capacity	Aware Basic interaction Very basic relationships Small chance of future communication	Low suffering	Medium pleasures
Significant Burden: based on Charlotte Wyatt	Aware Severe cognitive impairment—basic interaction, no relationships or communication	Moderate-severe suffering	Mild pleasures

These are not intended to capture the full details of each case—the complete survey can be viewed in online supplementary appendix F.

10.1136/medethics-2019-105639.supp1 Supplementary data

Each case was followed by the same set of questions. Participants first considered whether life was worth living for the infant by indicating the extent to which they agreed with three statements—‘Life has no benefit for this infant’; ‘Life is worse than death for this infant’ and ‘Life has benefit for this infant—it would be best for this infant to remain alive’ (7-point Likert scale: strongly agree–strongly disagree, 1–7). Participants next responded to a multiple-choice question on whether they believed it was morally obligatory to withdraw treatment, obligatory to continue or if either option was permissible. This distinction between the benefit (or lack of) of continued life and specific treatment decisions was made to ascertain the relationship between the two—for example, participants who believed life had some benefit might still believe it to be permissible to withdraw treatment.

In order to assess whether standards for treatment withdrawal would be different if considered from a personal perspective rather than impersonally, participants were then asked to indicate whether they would want treatment to be stopped if this was their own child. The final question aimed to assess the importance participants placed on parental autonomy: participants indicated on a 7-point Likert scale whether they believed treatment should be continued indefinitely if this was what the infant’s parents wished.

### Section 2

This section briefly addressed further ethical issues relating to withdrawal of treatment. Participants were asked which factors they believed to be most important in making treatment withdrawal decisions (options included the pain or pleasure the infant might experience; awareness; ability to interact, have relationships or communicate; survival time; potential for improvement; sanctity of human life; religious reasons; resource constraints and parental wishes).

Although participants were asked to ignore financial considerations throughout the earlier cases, the next question in this section directly addressed resource constraints. A brief explanation was given of finite medical resources, stating that continuing to treat these infants would result in other patients receiving less help. Participants were asked to indicate whether they believed resources should influence decisions (5-point scale, definitely yes - definitely no, 1–5).

Finally, participants were asked to rank who they believed should have the most influence on treatment withdrawal decisions in rare occasions of disagreement. Options given included parents, doctors, a hospital ethics committee, judges/the court and religious leaders.

### Section 3

In the last section, participants filled out the Oxford Utilitarianism Scale to investigate whether support for treatment withdrawal correlated with general utilitarian tendencies.^29^ They were asked questions from the Purity subscale of the Moral Foundations Scale to detect correlations between the value participants placed on concerns of religious norms, decency and purity and willingness to withdraw treatment.^30^ Endorsement of these statements has previously been associated with conservative morality.^31^

Demographic information obtained included age, gender, parental status, marital status, religiosity, nationality, ethnicity, highest completed education level and income category.

Statistical analysis was conducted using IBM SPSS Statistics version 25 for Mac. Descriptive and frequency statistics were used to describe the data. One sample t-tests were used to compare results against a neutral mid-point. Repeated measures analysis of variance (ANOVA) with a Greenhouse-Geisser correction and McNemar’s tests were used to analyse differences in responses between cases. Correlations between support of treatment withdrawal and predictor variables such as demographic information and responses to the Utilitarian Scale and Moral Foundations Scale were calculated using Spearman’s and Pearson’s correlation coefficients. P<0.05 was considered statistically significant.

## Results

One hundred and forty-nine participants took part in the survey. Nineteen were excluded for failing at least one of the three checks, leaving 130 valid participants. All participants were from the UK and 91.6% identified as White. Participants were aged between 18 and 75 years (M=37.5, SD=12.4). Seventy-two percent of participants were female and 28% were male. Fifty-nine per cent described themselves as having no religious affiliation or atheist, and on a scale of 1 (very religious) to 7 (not at all religious), the mean religiosity score was 6.08 (SD=1.59). Fifty-eight percent of participants were parents. Marital status, education and income levels were mixed.

### Agreement that life may be of NO benefit

**Figure 3:**
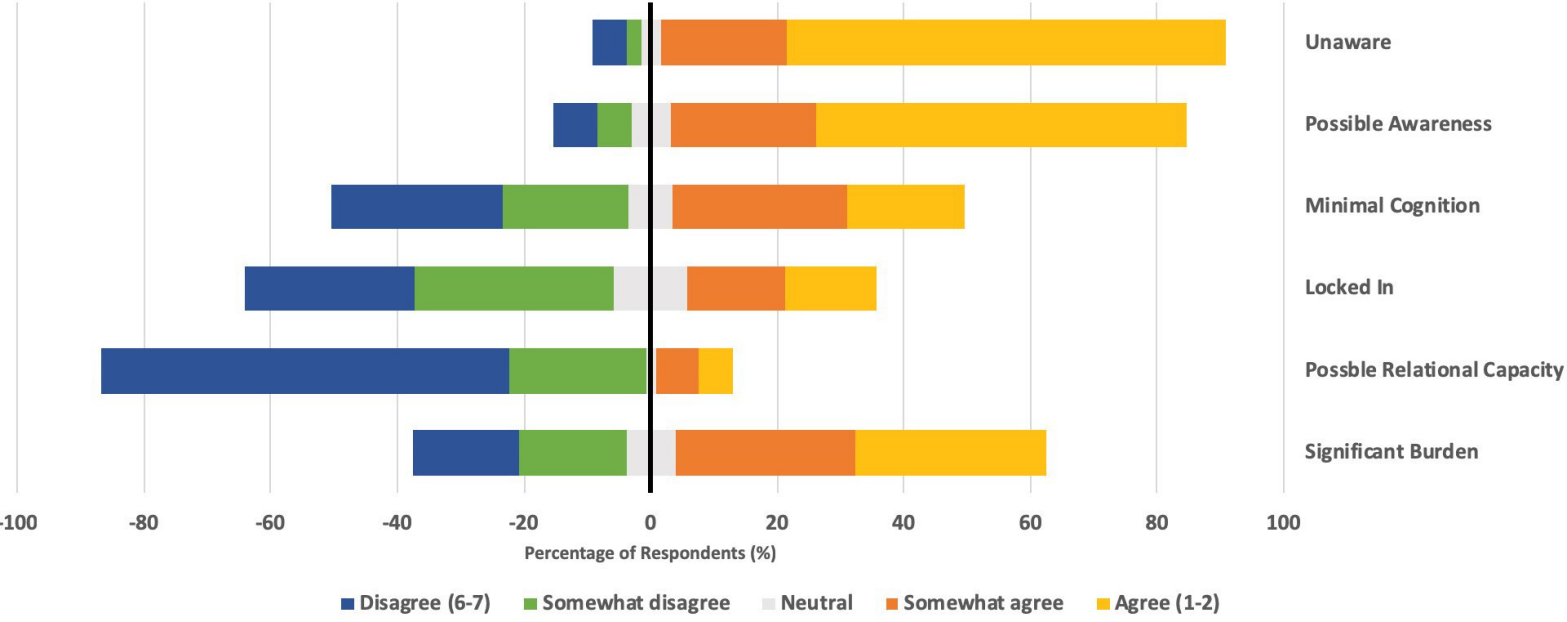
Distribution of values in response to the statement ‘Life has no benefit for this infant’.

Almost all respondents agreed that at some level of quality of life, life may be of no benefit or worse than death for an infant: 88% of participants agreed that life would be worse than death for at least one case and 94% agreed that life may be of no benefit. This endorsement varied significantly between cases, with the highest mean levels of agreement for *Unaware* (M=2.2, SD=1.4) compared with the lowest for *Possible Relational Capacity* (M=5.52, SD=1.4) (see online supplementary appendix A). The more severe the case, the more likely respondents were to agree life was of no benefit (*F*(4.224, 544.86)=138.63, p<0.001). This was so particularly for *Unaware* and *Possible Awareness,* where 89% and 81% of participants, respectively, agreed that life was of no benefit. In comparison, only 12% of participants agreed that life was of no benefit to the infant described in *Possible Relational Capacity* (figure 3).

### Intuitions on treatment withdrawal decisions

When asked to decide whether they believed that treatment should be continued, withdrawn or if either was permissible, participants significantly favoured withdrawal of treatment for the more severe cases and continuing treatment for the less severe cases. *Possible Relational Capacity* (the case where the infant had the least amount of pain and the most potential for future relationships) was the only case where a clear majority of participants (75%) believed treatment should be continued. In comparison, for *Minimal Cognition*, *Locked In* and *Significant Burden* (which had significantly more pain and distress), less than half of the participants believed treatment should be continued (table 2).

**Table 2:** Respondent intuitions on ethically permissible or obligatory treatment choices in the six cases

	Obligated to continue treatment (%)	Either is permissible (%)	Obligated to withdraw treatment (%)
Unaware	4.6	40.8	54.6
Possible Awareness	8.5	39.2	52.3
Minimal Cognition	35.4	52.3	12.3
Locked In	40.8	43.1	16.2
Possible Relational Capacity	75.4	20.8	3.8
Significant Burden	37.7	36.9	25.4

### Relationship between beliefs about the benefit of life and support for treatment withdrawal

**Figure 4:**
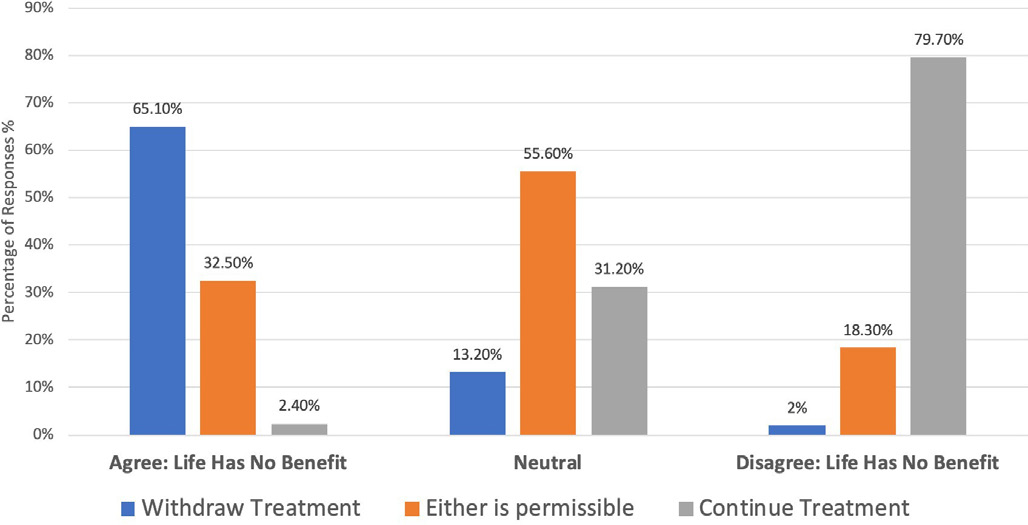
Aggregate data comparing responses to the statement ‘Life has no benefit’ with beliefs about morally correct treatment decisions.

Data across all cases demonstrated that in situations where respondents disagreed that ‘Life has no benefit’, a large proportion (80%) then thought it was ethically obligatory to continue treatment. In comparison, in situations where participants agreed that ‘Life has no benefit’, 65% thought that it was ethically obligatory to withdraw treatment and 33% indicated that either option was permissible (figure 4).

### Personal choice and parental autonomy

For every case, a higher proportion of participants would withdraw treatment if the child was their own than believed there was a moral obligation to withdraw treatment in a version of the same case where the child belonged to another family (p<0.05—online supplementary appendix B).

**Figure 5:**
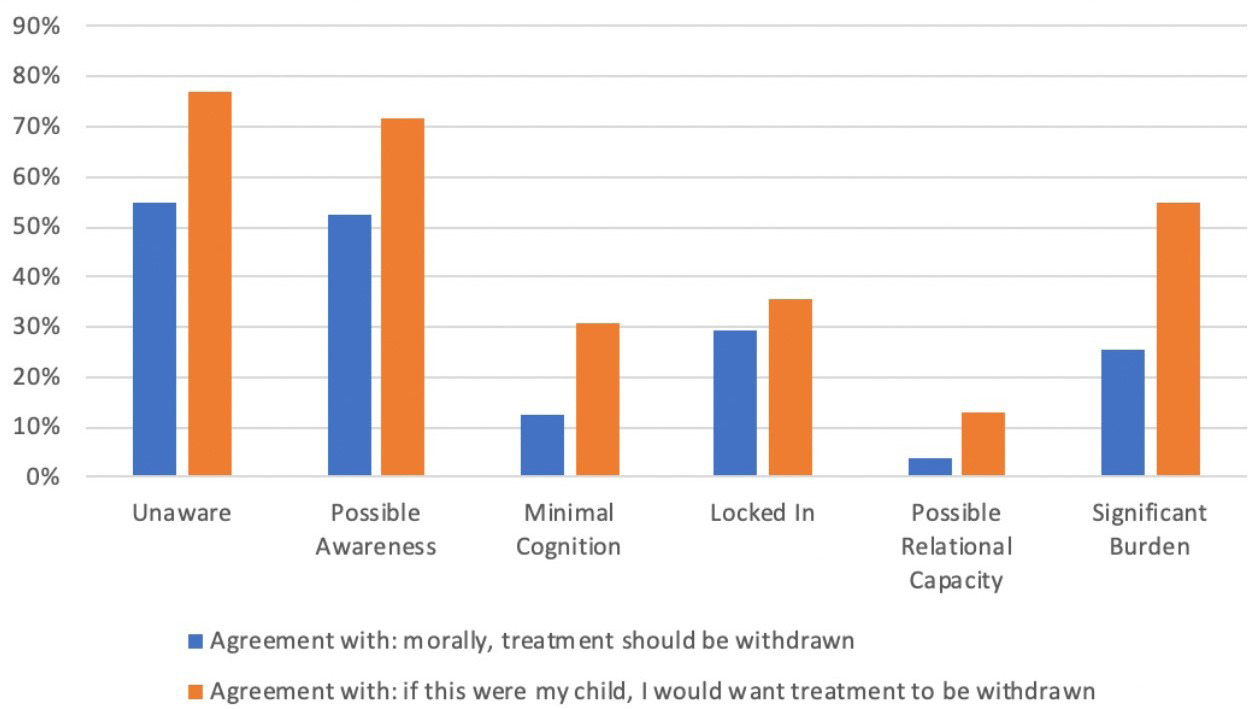
Comparison of the proportion of participants who would personally choose treatment withdrawal if it was their own child, with participants who agreed there was a general moral obligation to withdraw treatment.

However, participants did not in general support withdrawing treatment against parental opposition. The more severe the illness, the less likely participants were to agree with parents being allowed to indefinitely continue treatment for their child.^2^ In only one case did most participants believe that parents should not be allowed to request that treatment continue indefinitely: *Unaware* (52%). In the four least severe cases, only 12%–20% of participants supported treatment withdrawal against parental wishes (see online supplementary appendix C).

### Importance placed on factors associated with treatment withdrawal

Participants considered the presence of pain the most important factor (M=1.61) in deciding whether to withdraw treatment, followed closely by the presence of pleasure (M=1.96), awareness of surroundings (M=2.24) and potential for improvement (M=2.34). Survival time (M=3.42), the use of limited medical resources (M=4.38) and religion (M=6.18) were considered least important (see online supplementary appendix D).

Support for treatment withdrawal across all six cases was positively correlated with participants placing importance on the use of medical resources (r=0.286, p=0.001), the infant’s cognitive abilities (r=0.239, p=0.006), potential to have emotional relationships (r=0.197, p=0.025) and reliance on a feeding tube (r=0.188, p=0.033). In contrast, endorsement of treatment withdrawal was negatively correlated with placing importance on the pleasure the infant might experience, on parental choice and on the sanctity of human life (see online supplementary appendix E). Older people were less likely to support treatment withdrawal (r=0.197, p=0.043).

No correlations were found between participant gender, parental status, religiosity or the Moral Foundations Scale and support for treatment withdrawal. Endorsement of treatment withdrawal was also correlated with the Impartial Beneficence subscale of the Oxford Utilitarianism Scale (r=0.189, p=0.031) but not with the Instrumental Harm subscale (r=-.050, p=0.545). Disagreement with allowing parents to demand continued treatment was also correlated with the Impartial Beneficence subscale (r=0.271, p=0.002) and with endorsement of treatment withdrawal (r=0.691, p=0.001).

### Importance placed on resources

When asked about whether limited medical resources should influence treatment decisions, 42% of participants agreed that resources should be considered, while 40% disagreed and 18% were neutral. The Instrumental Harm subscale of the Oxford Utilitarianism Scale significantly correlated with endorsement of considering resources (Spearman’s Correlation Coefficient 0.291, p<0.001), but not the Impartial Beneficence subscale.

## Discussion

To our knowledge, this is the first study to examine public intuitions towards quality of life and treatment withdrawal decisions in paediatrics. It provides some important insights into the views of the UK general public on hypothetical versions of some highly controversial cases of disagreement between parents and doctors about treatment for a child. For three of the cases, a large majority of respondents agreed on whether life was worth living: for children who were unaware or possibly aware, 81% of respondents did not believe life was of benefit to the infant, while 86% of respondents believed that life *was* of benefit for a child who was severely disabled but had the potential for basic social relationships. Up to 50% of participants in each of the six cases believed that it was permissible to either continue or withdraw treatment. Participants did not generally support withdrawing treatment against parental objection: in five of six cases, a majority of participants indicated that treatment should be continued indefinitely for a child if parents wished this. The only case where participants believed parental wishes may be overruled was the case where the child was completely unaware.

### A life not worth living

These findings indicate that despite familiar controversies about judgements relating to quality of life, there may in fact be a level at which most people reach consensus that life is not worth living: one where cognition is so limited that the infant has no awareness of themselves or their surroundings, *even* if suffering is minimal. On the opposite end of the spectrum, a level of cognition that allows for basic relationships with parents and a small chance of future communication alongside the absence of pain and distress were defining features for a life good enough to be worth living. The remaining three cases, containing a mixture of benefits and suffering, divided opinion.

There were positive correlations between endorsement of treatment withdrawal across all cases and placing importance on the cognitive capacity and relational potential of the infant; those who valued the pleasure the infant might experience were less likely to endorse withdrawing treatment. Participants seemed to place most value on the objective goods of awareness and capacity for basic relationships when making this judgement. This might indicate implicit endorsement of an ‘objective list’ approach to well-being and the value of life. Rhoden and Robertson have argued that simple pleasure alone is a low bar to reach.^32 33^ It seems plausible that respondents who value pleasure highly will see life as worth living at a lower level of well-being compared with those who value cognition. This finding is concordant with a previous study on public attitudes towards treatment withdrawal in adults with disorders of consciousness, which found that the most important factors in these decisions were autonomy, presence of consciousness and ability to interact with others.^34^

Unsurprisingly, valuing the use of limited medical resources was also positively correlated with support of treatment withdrawal—it is likely that these respondents were influenced by distributive justice concerns, even when directed in the survey to disregard financial constraints. Negative correlations were found between valuing sanctity of life and support of treatment withdrawal. There were no direct correlations found with religiosity, though this may be because participants were generally non-religious.

### Attitudes towards treatment withdrawal compared with benefit of life

The study also assessed the difference between opinion on benefit of life and treatment withdrawal. As discussed earlier, the threshold for each has been theorised to lie at different levels of well-being, with a ‘threshold framework’ developed in response to the uncertainty involved in these decisions.^9^

Our findings seem to lend support to this threshold framework. Up to half (20%–52%) of respondents believed it was morally permissible to *either* withdraw or continue treatment in each case. We also noted that when participants believed that life had no benefit, 65% subsequently thought treatment should be withdrawn, while only 32% believed either was permissible. When participants believed life *was* of benefit, 80% subsequently believed treatment should continue and 18% thought either was permissible. Importantly, even when participants were not certain if life was of benefit, 55% still thought withdrawal was permissible.

Although we cannot deduce the reasons behind this uncertainty, it seems likely that there is a combination of doubt as to the morally correct option and a recognition that in some situations either option may be genuinely permissible. The latter seems to occur particularly when well-being is very close to the ‘zero-point’ of life having no benefit, and therefore it is very challenging to determine whether it is above, on or below that line—thus a more permissive approach accommodates this uncertainty.^4^ Ethically, such a view might seem problematic—it allows for some occasions of treatment withdrawal for infants who may have had a life of some small benefit and continuance for infants who may have a LNWL, thereby causing harm to these infants. However, given the significant moral and prognostic uncertainty, these kinds of harms are arguably impossible to avoid.

**Figure 6:**
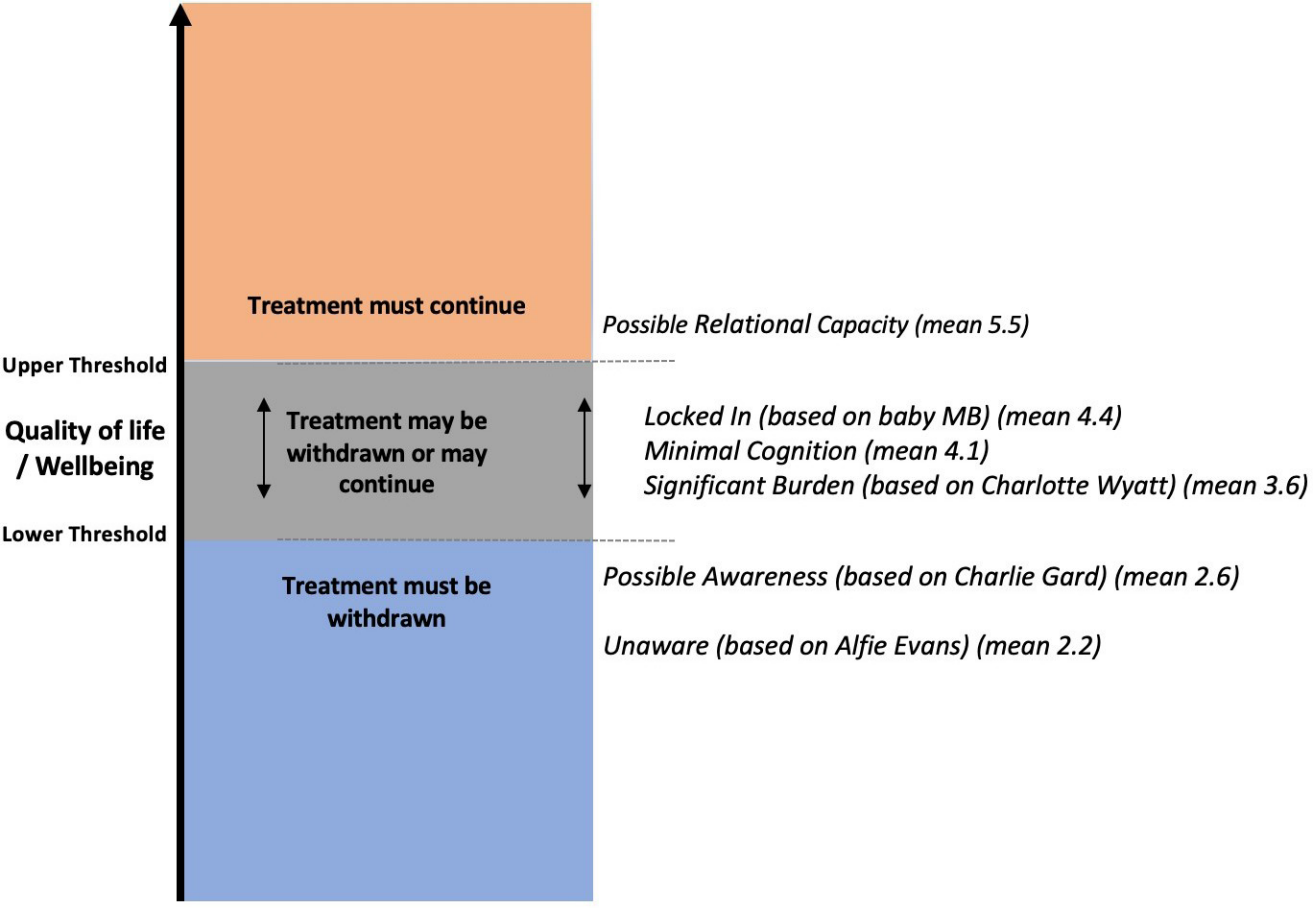
The Threshold Framework with the evaluations of the six cases by survey respondents displayed in order of agreement with the statement ‘Life is of no benefit to this infant’ (mean responses to Likert scale 1–7, where one is agree, four is neither agree nor disagree, and seven is disagree).

If we assess the survey cases using the Threshold View, it might imply that the scenarios where most respondents reach consensus fall outside the threshold: *Unaware* and *Possible Awareness* below the lower threshold, where the vast majority agree treatment should be withdrawn, and *Possible Relational Capacity* above the upper threshold, where the vast majority agree treatment should continue. It seems plausible to argue that the remaining cases that divided participant opinion might fall within the threshold, where it would be morally acceptable to either withdraw or continue treatment (figure 6).

The finding, in our study, that respondents did not generally agree with overriding the wishes of parents (who desired continuing life sustaining treatment indefinitely) confirmed one of our hypotheses and is supported by previous research.^22 35 36^ It is unsurprising that people in wider society tend to be reluctant to interfere with these parental medical choices. The prospect of a child dying is an awful one: parents have a high level of autonomy over other aspects of their children’s lives, and many may feel, reasonably, that as the people who know the child best and are tasked with caring for them, parents are best placed to make these decisions. However, there were limits to this. Our results suggest that when respondents believed treatment withdrawal to be the right choice, they tended to disagree with parental autonomy; when they believed it was permissible to withdraw *or* continue treatment, they shifted towards favouring parental autonomy and thus allowing treatment to continue.

A related concept has been proposed by ethicist Lynn Gillam: the Zone of Parental Discretion (ZPD). This is where clinicians may allow parents to make what clinicians believe to be suboptimal decisions for their child, as long as the ‘decisions do not involve probable harm to the child’, where harm is defined as a serious setback to overall well-being.^37^ These are often decisions where the parents hold a different conception of the best interests for their child to doctors and where prognostic uncertainty plays a role. It seems plausible that these three borderline cases may fall within the ZPD and thus that the deciding factor for treatment withdrawal in these cases could be parental preference. In contrast, the three cases where there was strong agreement about the treatment decision might represent situations where parental autonomy should be overridden: *Unaware* and *Possible Awareness* because treatment continuance may involve probable harm to the child and *Possible Relational Capacity* because treatment withdrawal may involve probable harm.

### Comparison of public attitudes to legal cases and medical guidelines

One aim of the survey was to compare public attitudes to controversial legal cases in order to gauge whether these court-ordered decisions are in line with prevailing moral beliefs. We note here that legal decisions are different to moral judgements—legal decisions often must consider other, non-moral factors relating to the law. However, in these paediatric treatment withdrawal cases, the legal precedent is that decisions must be based on the ethical principle of the best interests of the child. The ethical judgements of the public may therefore be compared with the legal decision.

Contrary to our hypothesis, public opinions were not markedly different to the legal outcomes (table 3). This suggests that the controversy about such verdicts, as played out in the media, may be influenced by vocal minority who are opposed to treatment withdrawal rather than being representative of population values. It is a reassuring finding that decisions of a democratically endorsed legal system seem in line with public values. It also may be useful in the development of further pathways to resolve disagreements and reduce escalation into costly, protracted court cases.

**Table 3:** Comparison of public attitudes to legal case judgements

	Legal outcome	Public attitudes from survey
Charlie Gard	Withdraw: *Justice Francis ruled that continuation of life would not be in Charlie’s best interests*.	Morally correct treatment choice (Possible Awareness case): Withdraw. *52% stated obligation to withdraw treatment. 39% stated permissible to withdraw or continue. 8% stated must continue*. If parents wanted treatment to be continued: Divided. *48% believed parents should not be allowed to demand continued treatment. 42% agreed parents should be allowed to demand that treatment continue*.
Alfie Evans	Withdraw: *ruled that it was unlawful to give treatment*.	Morally correct choice (Unaware case): Withdraw. *54% stated obligation to stop treatment. 41% stated permissible to withdraw or continue. 5% stated must continue*. If parents wanted treatment to be continued: Withdraw. *52% believed parents should not be allowed to demand continued treatment. 39% agreed parents should be allowed to demand that treatment continue*.
Baby MB	Continue: *maintain ventilation but withhold invasive procedures that might cause unnecessary additional distress*.	Morally correct treatment choice (Locked In Case): Continue. *41% stated obligation to continue treatment. 43% stated permissible to withdraw or continue*. If parents wanted treatment to be continued: Continue. *67% agreed parents should be allowed to demand continued treatment*.
Charlotte Wyatt	Continue: *although initially future life-sustaining treatment was withheld, Charlotte improved to the level described in the survey case scenario. It was then judged that mechanical ventilation should be provided in the event of future need*.	Morally correct treatment choice (Significant Burden Case): Divided. *38% stated obligation to provide life-sustaining treatment if needed. 37% stated permissible to provide or not. 25% stated obliged to withhold treatment*. If parents wanted treatment to be continued: Continue. *59% agreed parents should be allowed to continue treatment*.

However, we note the main limitation to this comparison is that the cases were considerably simplified for the purposes of the survey, and stipulated variables (such as the level of function) were often contested. For example, there was significant uncertainty and disagreement in the case of Charlie Gard about the level of pain experienced.

### Lack of priority given to resource constraints

Views were split on the importance that should be placed on limited medical resources: 40% believed scarce resources should influence treatment withdrawal decisions, while 40% believed they should not. This reflects previous surveys of public attitudes, which have demonstrated an aversion to using cost-benefit analysis to make decisions.^34^ Perhaps this seems too detached or impersonal or unfair to withdraw treatment based on quality of life. It is also possible that participants did not entirely comprehend the magnitude of costs involved in providing intensive care treatment, and the consequences of this to other patients. It is challenging to prioritise unknown future patients who might be negatively impacted by providing expensive treatment to a known infant, with little temporal immediacy or proximity to the decision.

However, it does not necessarily follow that we must ignore resources: this is arguably incompatible with a publicly funded healthcare system. Instead, the significance of this result may be in highlighting the significant gap between the analysis of medical ethicists, who often believe resources and distributive justice to be important,^4^ and the views of the general public. Importantly, the finding that respondents who placed value on resources were more likely to endorse treatment withdrawal may suggest that drawing greater attention to distributive justice concerns could sway public opinion. Resource constraints are becoming critical to almost every aspect of medicine and thus this is an area where public education campaigns could be valuable.

### Limitations

This study had several limitations. It was administered through an online platform which necessarily means participants completed the survey in an uncontrolled environment. The sample size of 130 was relatively small and though it was powered to detect effects in the main analysis, it may not be able to detect small subgroup differences. There was a higher proportion of white and female respondents than the UK general population, and the responses may not be generalisable to the wider public in the UK or in other countries.

There were also significant numbers of neutral responses, and it is unclear whether this represents genuine uncertainty in the face of challenging questions, if participants thought it was implausible or too difficult to judge an infant’s quality of life or whether they fully understood what the medical conditions were like. It is difficult to convey complex information in survey form, and scenarios had to be simplified in order to isolate factors that might influence judgements. Real cases involve significantly more background information and uncertain prognostic variables which may shape decisions, and a short survey is unlikely to give enough time and information to reflect on whether a particular life is worth living. The construction of the cases was limited to the facts described in the legal judgements.

## Conclusion

To our knowledge, this empirical study is the first to gather public views on conceptions of a LNWL in relation to paediatric treatment withdrawal, with focus on some of the most divisive UK legal cases. These decisions happen frequently in hospital intensive care units and disagreements are likely to increase with medical technology and public awareness of treatment options. Having comprehensive policy to inform these situations is necessary to reduce both unnecessary medical treatment and legal battles.

Although public views cannot give direct answers to normative questions about how to evaluate the benefits and burdens of a life, public opinion is still of ethical interest: to analyse and compare with theoretical principles to achieve coherent, practical conclusions (a process of reflective equilibrium) and to demonstrate any significant gaps between public views and those of ethicists, particularly in an area relevant to the publicly-funded healthcare system. Future empirical studies with a larger and more diverse population of participants could yield more generalisable results, while similar studies could be undertaken to examine the attitudes of health professionals and parents of unwell children in these situations. Future ethical analysis could work towards further refining and achieving coherence between varying conceptions of a life worth living and using this to construct a practical framework to inform health policy.

Our study has demonstrated that there may be a certain level of well-being at which most people agree life is not worth living. Decisions to withdraw treatment were associated with placing value on future abilities to learn and have basic relationships. This suggests that infants with very severe cognitive impairment are most likely to be regarded as falling below the level of a life worth living. Participants placed significant importance on the capacity to form basic social relationships in a life worth living. The high levels of support for permissible treatment withdrawal and parental autonomy in the more divisive cases may lend support to practical frameworks like Wilkinson’s Threshold View^9^ and Gillam’s ZPD,^37^ as well as providing further insight into where these thresholds might be set when constructing more specific guidelines. It is widely assumed that such decisions are highly controversial. Importantly, our findings suggest that there is wide acceptance for at least the permissibility of withdrawal of treatment across a range of cases.

## Footnotes

It must be emphasised that this does not necessarily lead to a conclusion that it is right to kill those whose lives are believed to be not worth living, but to say that to continue to live is no longer of benefit to them. Though some might argue for euthanasia in this context, here we instead concentrate on the more widely accepted implication that if a life is not worth living, life sustaining treatment may not, or perhaps should not, be continued.

A repeated measures ANOVA with a Greenhouse-Geisser correction determined that mean endorsement of parental autonomy differed statistically significantly between cases: F(3.944, 508.81)=52.76, p<0.001).
