# Supplementary material for: Worth living or worth dying? The views of the general public about allowing disabled children to die
Source: J Med Ethics. 2019 Oct 15;46(1):7–15. doi: 10.1136/medethics-2019-105639 (PMC6984061; doi:10.1136/medethics-2019-105639)
Supplement: Supplementary data [file medethics-2019-105639supp001.pdf]

Appendix

Appendix A.

Level of agreement with statements about the benefit of life for severely ill infants (as rated on a Likert scale 1-7, where 1 is agree and 4 is neither agree nor disagree, mean (sd))

	Unaware	Possible Awareness	Minimal Cognition	Locked In	Possible Relational Capacity	Significant Burden
**'Life is worse than death for this infant'**	3.14 (1.6)	2.81 (1.36)	4.37 (1.6)	4.33 (1.5)	5.52 (1.4)	3.56 (1.6)
**'Life has no benefit for this infant'**	2.2 (1.4)	2.58 (1.5)	4.11 (1.7)	4.43 (1.66)	5.53 (1.46)	3.58 (1.67)
**'Life has benefit – best for this infant to remain alive'**	5.35 (1.3)	5.13 (1.3)	3.79 (1.6)	3.75 (1.5)	2.42 (1.3)	4.46 (1.5)

Appendix B.

Personal choice compared to general obligation to withdraw treatment

For every case the number of participants who would withdraw treatment if the child was their own was greater than the number of participants who said there was a moral obligation to withdraw treatment. McNemar's test determined that there was a statistically significant difference in the proportions of participants who responded to the two questions. For all cases except case 5, a McNemar's test with continuity correction was used, $p<.001$. For case 5, an exact McNemar's test was used, $p=.002$. Case 1: $\chi2(1) = 22.400$, $p < .001$, case 2: $\chi2(1) = 15.568$, $p <.001$, case 3: $\chi2(1) = 20.346$, $p <.001$, case 4: $\chi2(1) = 18.581$, $p <.001$, case 6: $\chi2(1) = 32.595$, $p <.001$.

Appendix C.

Means (mean (sd)) and distributions of frequencies of agreement/disagreement with a statement that parents should be allowed to continue treatment indefinitely for their child, across the 6 cases.

		Unaware	Possible Awareness	Minimal Cognition	Locked In	Basic Relational Capacity	Significant Burden
Mean agreement		4.18 (1.8)	4.05 (1.7)	3.05 (1.57)	3.02 (1.55)	2.39 (1.54)	0.48 (1.78)
Frequencies of agreement	Agree (1-3)	39.2%	42.3%	68.5%	66.2%	79.2%	58.5%
	Neutral (4)	8.5%	10%	10.8%	10%	8.5%	7.7%
	Disagree (5-7)	52.3%	47.7%	20.8%	23.8%	12.3%	16.9%

Appendix D.

Public endorsement of factors associated with treatment withdrawal decisions, ordered from most to least important.

Values / factors	Mean	Median
Pain	1.61	1
Pleasure / joy	1.96	2
Awareness of surroundings	2.24	2
Potential for improvement, even if extremely small	2.34	2
Ability to interact	2.54	2
Cognition – learning, communication	2.68	2
Emotional relationship potential	2.77	3
Parental choice	2.95	3
Sanctity of human life	3.15	3
Reliance on a breathing machine/ feeding tube	3.29	3
Survival time estimate	3.42	3
Using up medical resources that could be used to help other patients	4.38	4
Teachings of my religion	6.18	7

Appendix E.

Importance of factors correlated with endorsement of treatment withdrawal across all six cases.

Values / Factors	Pearson Correlation Co-efficient	Statistical Significance (2-tailed)
Statistically Significant Positive Correlations		
**Using up medical resources that could be used to help other patients**	.286	.001
**Cognition – learning, communication**	.239	.006
**Emotional relationship potential**	.197	.025
**Reliance on a breathing machine/ feeding tube**	.188	.033
**Survival time estimate**	.183	.037
Statistically Significant Negative Correlations		
**Parental choice**	-.633	.000
**Pleasure / joy**	-.320	.000
**Sanctity of human life**	-.408	.000
**Age**	-.179	.043

Appendix F. Survey

You will be presented with descriptions of several severe medical conditions in infants. Some of the situations are based on real-life cases. We will then ask you questions about your view about life in these conditions. Doctors and family members sometimes have to make these kinds of difficult decisions about when to stop treatament for seriously ill infants, and we are investigating how people think about these dilemmas.

In answering these questions, please set aside considerations relating to the current law in your country. We are only interested in what you think is morally correct, and about what you think should be done in such difficult circumstances.

Please read the text and questions carefully and respond as honestly as you can. Later on, there will be control questions designed to make sure that you are reading the texts carefully enough. If you answer these control questions incorrectly, you might be excluded from the study.

→

Section A. Cases

All the cases we will now describe involve a sick infant who is being kept alive in the hospital intensive care unit by mechanical ventilation (a breathing machine) and artificial nutrition and hydration (being fed through a tube). If the breathing machine and feeding tube were withdrawn, all possible care would be taken to minimise pain and suffering, and the infant would soon die.

Some of these cases may appear similar, so do pay attention to the specific details of each individual case.

In answering these questions, **please set aside considerations of the effect of decisions on the family and the cost of treatment.** We will ask you about these further issues later on.

→

Please consider the following case:

An 18 month old baby in hospital has a disorder which means they are reliant on a breathing machine and feeding tube to survive.

- **Physical and sensory abilities:** the infant can interact with their surroundings by moving their eyes and arms and feeling the sensation of touch.
- **Mental abilities**: the infant has some limited mental abilities. They can experience pleasure from seeing and hearing their parents and watching TV programs. In future, they may be able to understand language at about the level of a two year old and there is a small chance they will be able to communicate.
- **Suffering:** the infant is experiencing minor pain and discomfort from being in hospital.
- **Future prospects:** if treatment is continued, the infant is likely to survive for a few years at this level of disability. There is a small chance they will be able to communicate in future.

Please indicate to what extent you agree or disagree with the following statements:

	Strongly agree	Agree	Somewhat agree	Neither agree nor disagree	Somewhat disagree	Disagree	Strongly disagree
Life has no benefit for this infant	○	○	○	○	○	○	○
Life is worse than death for this infant	○	○	○	○	○	○	○
Life has benefit for this infant - it would be best for this infant to remain alive	○	○	○	○	○	○	○

Please select the statement that you most agree withregarding treatment in this case

- ○ Treatment *should* be continued – it would be morally wrong to stop treating this infant
- ○ It is morally acceptable both to stop treatment and to continue it – there is no moral obligation to do one of the two
- ○ Treatment *should* be stopped and the infant allowed to pass away – it would be wrong to keep this infant alive in these conditions

Please indicate to what extent you agree or disagree that:

	Strongly agree	Agree	Somewhat agree	Neither agree nor disagree	Somewhat disagree	Disagree	Strongly disagree
If this were my own child, I would want treatment to be stopped	○	○	○	○	○	○	○

We now want to ask you what you think **the role of the infant's parents should be** in making such decisions. In particular, do you agree that:

	Strongly agree	Agree	Somewhat agree	Neither agree nor disagree	Somewhat disagree	Disagree	Strongly disagree
If the parents of this infant wanted treatment to be continued indefinitely, this should be allowed	○	○	○	○	○	○	○

→

Please consider the following case:

A baby has a rare disorder that means that by 6 months of age they are being kept alive in hospital with a breathing machine and a feeding tube.

- **Physical and sensory abilities:** the infant is completely paralysed (cannot move their arms or legs or face at all) and deaf.
- **Mental abilities:** the infant has limited mental abilities. They are unlikely to be aware of their surroundings. They are extremely unlikely to ever respond, interact or communicate.
- **Suffering:** the infant may be suffering pain and discomfort through procedures involved with being in hospital, such as suction from the breathing machine.
- **Future prospects**: there is almost no chance of improvement, but the infant is likely to survive at this level of disability for a few years if treatment is continued.

Please indicate to what extent you agree or disagree with the following statements:

	Strongly agree	Agree	Somewhat agree	Neither agree nor disagree	Somewhat disagree	Disagree	Strongly disagree
Life has no benefit for this infant	○	○	○	○	○	○	○
Life is worse than death for this infant	○	○	○	○	○	○	○
Life has benefit for this infant - it would be best for this infant to remain alive	○	○	○	○	○	○	○

Please select the statement that you most agree with regarding treatment in this case

- ○ Treatment *should* be continued – it would be morally wrong to stop treating this infant
- ○ It is morally acceptable both to stop treatment and to continue it – there is no moral obligation to do one of the two
- ○ Treatment *should* be stopped and the infant allowed to pass away – it would be wrong to keep this infant alive in these conditions

Please indicate to what extent you agree or disagree that:

	Strongly agree	Agree	Somewhat agree	Neither agree nor disagree	Somewhat disagree	Disagree	Strongly disagree
If this were my own child, I would want treatment to be stopped	○	○	○	○	○	○	○

We now want to ask you what you think **the role of the infant's parents should be** in making such decisions. In particular, do you agree that:

	Strongly agree	Agree	Somewhat agree	Neither agree nor disagree	Somewhat disagree	Disagree	Strongly disagree
If the parents of this infant wanted treatment to be continued indefinitely, this should be allowed	○	○	○	○	○	○	○

→

Please consider the following case:

A baby develops paralysis so by the time they are 18 months old, they can only move their eyes. The infant requires a breathing machine and a feeding tube to be kept alive.

- **Physical and sensory abilities**: the infant is paralysed (they cannot move their arms or legs or face) but can move their eyes. They can see, hear and feel the sensation of touch.
- **Mental abilities:** the infant is mentally like a normal toddler - they are aware of their surroundings and are likely to get pleasure from seeing their parents.
- **Suffering:** the infant is feeling pain and discomfort from the breathing machine and other procedures like getting blood taken. They are likely to be distressed from being unable to move or communicate. They cannot cry, breathe or swallow.
- **Future prospects:** if they continue to live, the infant may be able to understand some language and have thoughts like a child of their age, but will not be able to communicate or move. They will continue to undergo procedures that will cause pain and distress. The infant is likely to survive for a few years if treatment is continued.

Please indicate to what extent you agree or disagree with the following statements:

	Strongly agree	Agree	Somewhat agree	Neither agree nor disagree	Somewhat disagree	Disagree	Strongly disagree
Life has no benefit for this infant	○	○	○	○	○	○	○
Life is worse than death for this infant	○	○	○	○	○	○	○
Life has benefit for this infant - it would be best for this infant to remain alive	○	○	○	○	○	○	○

Please select the statement that you most agree with regarding treatment in this case

- ○ Treatment *should* be continued – it would be morally wrong to stop treating this infant
- ○ It is morally acceptable both to stop treatment and to continue it – there is no moral obligation to do one of the two
- ○ Treatment *should* be stopped and the infant allowed to pass away – it would be wrong to keep this infant alive in these conditions

Please indicate to what extent you agree or disagree that:

	Strongly agree	Agree	Somewhat agree	Neither agree nor disagree	Somewhat disagree	Disagree	Strongly disagree
If this were my own child, I would want treatment to be stopped	○	○	○	○	○	○	○

Withdrawing treatment from patients with these conditions is extremely controversial. It is therefore very important that the answers to these surveys reflect people's true values. This is a test question to check that you are reading the instructions.

	Strongly agree	Agree	Somewhat agree	Neither agree nor disagree	Somewhat disagree	Disagree	Strongly disagree
If you are reading this, please select disagree	○	○	○	○	○	○	○

We now want to ask you what you think **the role of the infant's parents should be** in making such decisions. In particular, do you agree that:

	Strongly agree	Agree	Somewhat agree	Neither agree nor disagree	Somewhat disagree	Disagree	Strongly disagree
If the parents of this infant wanted treatment to be continued indefinitely, this should be allowed	○	○	○	○	○	○	○

→

Please consider the following case:

A 6 month old baby in hospital has a disorder which means they are reliant on a breathing machine and a feeding tube to survive.

- **Physical and sensory abilities:** the infant is blind and deaf.
- **Mental abilities:** the infant has significant brain damage but is aware of its surroundings. The infant can feel the sensation of touch and experience mild pleasures like being bathed and held. They are extremely unlikely to ever be able to respond, interact or communicate.
- **Suffering**: the infant is experiencing minimal pain and discomfort.
- **Future prospects**: there is no hope of improvement, but the infant is likely to survive at this level of disability for a few years if treatment is continued.

Please indicate to what extent you agree or disagree with the following statements:

	Strongly agree	Agree	Somewhat agree	Neither agree nor disagree	Somewhat disagree	Disagree	Strongly disagree
Life has no benefit for this infant	○	○	○	○	○	○	○
Life is worse than death for this infant	○	○	○	○	○	○	○
Life has benefit for this infant - it would be best for this infant to remain alive	○	○	○	○	○	○	○

Please select the statement that you most agree with regarding treatment in this case

- ◯ Treatment *should* be continued – it would be morally wrong to stop treating this infant
- ◯ It is morally acceptable both to stop treatment and to continue it – there is no moral obligation to do one of the two
- ◯ Treatment *should* be stopped and the infant allowed to pass away – it would be wrong to keep this infant alive in these conditions

Please indicate to what extent you agree or disagree that:

	Strongly agree	Agree	Somewhat agree	Neither agree nor disagree	Somewhat disagree	Disagree	Strongly disagree
If this were my own child, I would want treatment to be stopped	◯	◯	◯	◯	◯	◯	◯

We now want to ask you what you think **the role of the infant's parents should be** in making such decisions. In particular, do you agree that:

	Strongly agree	Agree	Somewhat agree	Neither agree nor disagree	Somewhat disagree	Disagree	Strongly disagree
If the parents of this infant wanted treatment to be continued indefinitely, this should be allowed	◯	◯	◯	◯	◯	◯	◯

Please consider the following case:

A baby is born very prematurely (early) which left them with severe impairments. They are now 2 years old and have spent almost all of their life in hospital. The infant often suffers infections and illnesses requiring invasive, painful treatments. **Unlike the other cases we describe, the infant can currently breathe on its own. However, in the likely event of a future severe infection, a breathing machine is likely to be needed and may or may not sustain their life until the next illness.**

- **Physical and sensory abilities:** the infant has lung disease which means they require oxygen through a tube in their nose, and severe spastic quadriplegia (cerebral palsy) which means they cannot use their legs or arms. The infant has impaired hearing and vision. They are fed through a tube but can taste some food.
- **Mental abilities:** the infant has severe brain damage and suffers seizures, but can react to loud noises and colourful objects at about the level of a 1 month old child. They are very unlikely to ever understand language or communicate.
- **Suffering**: the infant has painful limb deformities and clearly experiences significant pain and distress through medical procedures.
- **Future prospects:** the chances of any further improvement are small but they may survive at this level of disability for 5 to 10 years.

Please indicate to what extent you agree or disagree with the following statements:

	Strongly agree	Agree	Somewhat agree	Neither agree nor disagree	Somewhat disagree	Disagree	Strongly disagree
Life has no benefit for this infant	○	○	○	○	○	○	○
Life is worse than death for this infant	○	○	○	○	○	○	○
Life has benefit for this infant - it would be best for this infant to remain alive	○	○	○	○	○	○	○

Please select the statement that you agree with the most regarding treatment in this case

- ○ When the infant next experiences a severe illness, treatment like a breathing machine *should* be provided – it would be morally wrong to not treat this infant
- ○ When the infant next experiences a severe illness, it is morally acceptable both to provide or not provide a breathing machine – there is no moral obligation to do one of the two
- ○ When the infant next experiences a severe illness, treatment like a breathing machine *should not* be provided - it would be wrong to start invasive treatment in these conditions

Please indicate to what extent you agree or disagree that:

	Strongly agree	Agree	Somewhat agree	Neither agree nor disagree	Somewhat disagree	Disagree	Strongly disagree
If this were my own child, I would want treatment to be withheld in the event of a future severe illness	○	○	○	○	○	○	○

We now want to ask you what you think **the role of the infant's parents should be** in making such decisions. In particular, do you agree that:

	Strongly agree	Agree	Somewhat agree	Neither agree nor disagree	Somewhat disagree	Disagree	Strongly disagree
If the parents of this infant wanted treatment to be continued indefinitely, this should be allowed	○	○	○	○	○	○	○

Please consider the following case:

A baby is born healthy but does not develop as normal, and by 6 months of age is diagnosed with a severe brain disorder. The infant is being kept alive in hospital with a breathing machine and a feeding tube.

- **Physical and sensory abilities:** the infant is blind and deaf. They cannot feel anything.
- **Mental abilities:** the infant has almost no mental abilities - they essentially have no consciousness and no awareness of their surroundings. They cannot think or form emotions and are similar to a person in a coma.
- **Suffering**: the infant is very unlikely to be experiencing any pain or pleasure.
- **Future prospects:** there is no hope of improvement, but the infant is likely to survive at this level of disability for a few years if treatment is continued.

Please indicate to what extent you agree or disagree with the following statements:

	Strongly agree	Agree	Somewhat agree	Neither agree nor disagree	Somewhat disagree	Disagree	Strongly disagree
Life has no benefit for this infant	○	○	○	○	○	○	○
Life is worse than death for this infant	○	○	○	○	○	○	○
Life has benefit for this infant - it would be best for this infant to remain alive	○	○	○	○	○	○	○

Please select the statement that you most agree with regarding treatment in this case

- ◯ Treatment *should* be continued – it would be morally wrong to stop treating this infant
- ◯ It is morally acceptable both to stop treatment and to continue it – there is no moral obligation to do one of the two
- ◯ Treatment *should* be stopped and the infant allowed to pass away – it would be wrong to keep this infant alive in such conditions

Please indicate to what extent you agree or disagree that:

	Strongly agree	Agree	Somewhat agree	Neither agree nor disagree	Somewhat disagree	Disagree	Strongly disagree
If this were my own child, I would want treatment to be stopped	◯	◯	◯	◯	◯	◯	◯

We now want to ask you what you think **the role of the infant's parents should be** in making such decisions. In particular, do you agree that:

	Strongly agree	Agree	Somewhat agree	Neither agree nor disagree	Somewhat disagree	Disagree	Strongly disagree
If the parents of this infant wanted treatment to be continued indefinitely, this should be allowed	◯	◯	◯	◯	◯	◯	◯

Which of the following statements is **true** about the infant in the case you **just** read above and answered questions about?

- ◯ The infant has no consciousness
- ◯ The infant can move its arms
- ◯ The infant gets pleasure from hearing its parents
- ◯ The infant can breathe on its own

→

Section B: General Questions

In considering whether treatment should be stopped or continued for these infants, how much importance do you place on the following factors:

	Extremely important	Very important	Moderately important	Neutral	Slightly important	Low importance	Not at all important
The pain the infant is suffering	○	○	○	○	○	○	○
The pleasure and joy the infant might experience	○	○	○	○	○	○	○
Whether the infant is aware of its surroundings	○	○	○	○	○	○	○
The infant's ability to interact with others and their environment	○	○	○	○	○	○	○
The infant's ability to have significant emotional relationships e.g. with parents and siblings	○	○	○	○	○	○	○
The infant's potential future mental abilities (to communicate, learn)	○	○	○	○	○	○	○
The fact that they rely on a breathing machine and feeding tube to live	○	○	○	○	○	○	○
The length of time the infant is expected to survive, even if unconscious and unaware	○	○	○	○	○	○	○

The potential, even if extremely small, for further significant improvement	○	○	○	○	○	○	○
The sanctity of human life	○	○	○	○	○	○	○
The teachings of my religion	○	○	○	○	○	○	○
Treatment using up medical resources that could be used to help other patients	○	○	○	○	○	○	○
What the parents want	○	○	○	○	○	○	○

Are there any other factors you would consider when deciding whether to stop treatment? If so, please enter them here (optional).

Medical resources are limited and treatment for infants with these conditions is extremely expensive. This means that **treating seriously ill infants means other patients will receive less help.** Considering these resource constraints, do you think financial cost should influence decisions about stopping treatment?

Definitely yes	Probably yes	Neutral	Probably not	Definitely not
○	○	○	○	○

→

For most of these difficult cases where a decision must be made about whether to stop treatment, parents and doctors agree on what to do. Very occasionally there is disagreement. **When there is disagreement, who do you think should have more influence on the decision?**

Please rank each group, with **1 being the people you believe should have the most influence on the decision and 8 the people who should have the least influence on the decision**. Type your ranking into the text box next to each group.
If you are leaving the field 'other' blank, type 8 into this box.

☐ Judges / the court

☐ Doctors

☐ Other - specify ________

☐ Other family members

☐ A hospital ethics committee

☐ A medical ethics expert

☐ Parents

☐ A religious leader

Section C : Scales

Please read the following statements. When you decide whether something is right or wrong, to what extent are the following considerations relevant to your thinking?

	Not at all relevant (This consideration has nothing to do with my judgments of right and wrong)	Not very relevant	Slightly relevant	Somewhat relevant	Very relevant	Extremely relevant (This is one of the most important factors when I judge right and wrong)
Whether or not someone violated standards of purity and decency	○	○	○	○	○	○
Whether or not someone did something disgusting	○	○	○	○	○	○
Whether or not someone acted in a way that God would approve of	○	○	○	○	○	○

Please read the following list of statements and indicate how much you agree or disagree with each statement.

	Strongly disagree	Moderately disagree	Slightly disagree	Slightly agree	Moderately agree
People should not do things that are disgusting, even if no one is harmed	○	○	○	○	○
I would call some acts wrong on the grounds that they are unnatural	○	○	○	○	○
Chastity is an important and valuable virtue	○	○	○	○	○

Please read the following list of statements and indicate how much you agree or disagree with each statement.

"Please select the second option from the bottom."

"From a moral point of view, we should feel obliged to give one of our kidneys to a person with kidney failure since we don't need two kidneys to survive, but really only one to be healthy."

"It is morally right to harm an innocent person if harming them is a necessary means to helping several other innocent people."

"If the only way to ensure the overall well-being and happiness of the people is through the use of political oppression for a short, limited period, then political oppression should be used."

"Sometimes it is morally necessary for innocent people to die as collateral damage—if more people are saved overall."

"From a moral perspective, people should care about the well-being of all human beings on the planet equally; they should not favor the well-being of people who are especially close to them either physically or emotionally."

"If the only way to save another person's life during an emergency is to sacrifice one's own leg, then one is morally required to make this sacrifice."

"It is morally wrong to keep money that one doesn't really need if one can donate it to causes that provide effective help to those who will benefit a great deal."

"It is just as wrong to fail to help someone as it is to actively harm them yourself."

"It is permissible to torture an innocent person if this would be necessary to provide information to prevent a bomb going off that would kill hundreds of people."

Section D : Demographics

We are now in the final part of this study.

What is your gender?

- ○ Male
- ○ Female
- ○ Other- feel free to specify or leave blank as you wish

What is your age?

Are you a parent?

- ○ Yes
- ○ No

What is your marital status?

- ○ Single
- ○ Married
- ○ De facto

How religious do you consider yourself to be?

Extremely religious						Not at all religious
○	○	○	○	○	○	○

What is your religion?

- Church of England
- Catholic
- Christian - other
- Islam / Muslim
- Sikh
- Jewish
- Buddhist
- Hindu
- No religious affiliation
- Atheist
- Other - specify

What is your nationality?

What is your ethnicity?

- Arab
- Asian: Bangladeshi
- Asian: Chinese
- Asian: Indian
- Asian: Pakistani
- Asian: other background
- Black: African
- Black: Carribbean
- Black: other background
- Mixed: White and Asian
- Mixed: White and Black African
- Mixed: White and Black Caribbean
- Mixed: other background
- White: British
- White: Irish
- White: Gypsy, Roma or Irish Traveller
- White: other background
- Any other ethnic background (please specify)
- Prefer not to say

What is the highest level of education you have completed?

- ◯ Less than secondary school (high school)
- ◯ Secondary school (GCSEs/O levels)
- ◯ Sixth form/College (A levels)
- ◯ University - Undergraduate degree
- ◯ University - Postgraduate degree

Please choose the category that describes the total amount of income you earned during the last year. Consider all forms of income, including salaries, tips, interest and dividend payments, scholarship support, student loans, parental support, social security, alimony, and child support, and others.

- ◯ Under £5000
- ◯ £5000 - £10,000
- ◯ £10,001 - £15,000
- ◯ £15,0001 - £25,000
- ◯ £25,001 - £35,000
- ◯ £35,001 - £50,000
- ◯ £50,001 - £100,000
- ◯ £100,001 - £200,000
- ◯ More than £200,000

Please enter your Prolific ID (it can be found at the top of this webpage or when going into your account information).

Do you think the reward for this task is fair?

- ◯ Yes
- ◯ No

Any other final comments or feedback? (optional)
